# Acute Dysphagia Following Reperfusion Therapies: A Prospective Pilot Cohort Study

**DOI:** 10.1007/s00455-023-10599-6

**Published:** 2023-06-28

**Authors:** Ellie Minchell, Anna Rumbach, Anna Farrell, Clare L. Burns, Andrew Wong, Emma Finch

**Affiliations:** 1 School of Health and Rehabilitation Sciences, The University of Queensland, Brisbane, Australia; 2 Royal Brisbane and Women’s Hospital, Metro North Health, Queensland Health, Brisbane, Australia; 3 Centre for Functioning and Health Research, Metro South Health, Queensland Health, Brisbane, Australia; 4 Research and Innovation, West Moreton Health, Queensland Health, Ipswich, Australia; 5 Princess Alexandra Hospital, Metro South Health, Queensland Health, Brisbane, Australia; 6 School of Medicine, The University of Queensland, Brisbane, Queensland Australia

**Keywords:** Dysphagia, Endovascular thrombectomy, Thrombolysis, Reperfusion therapies, Stroke

## Abstract

Dysphagia is a well-documented sequela of stroke. Recent advancements in medical treatments for stroke include reperfusion therapies (endovascular thrombectomy (EVT) and thrombolysis). As outcomes following reperfusion therapies are typically measured via general functional scales, the pattern and progression of acute dysphagia following reperfusion therapies is less known. To determine the progression of acute dysphagia (0–72 h) following reperfusion therapies and relationships between various stroke parameters and dysphagia, twenty-six patients were prospectively recruited across two EVT and thrombolysis centres in Brisbane, Australia. Dysphagia was screened via the Gugging Swallowing Screen (GUSS) at the bedside at three timepoints: 0–24 h, 24–48 h, and 48–72 h post-reperfusion therapies. Across three groups (EVT only, thrombolysis only, or both), the incidence of any dysphagia within the first 24 h of reperfusion therapy was 92.31% (*n* = 24/26), 91.30% (*n* = 21/23) by 48 h, and 90.91% (*n* = 20/22) by 72 h. Fifteen patients presented with severe dysphagia at 0–24 h, 10 at 24–48 h, and 10 at 48–72 h. Whilst dysphagia was not significantly correlated to infarct penumbra/core size, dysphagia severity was significantly related to the number of passes required during EVT (*p* = 0.009).Dysphagia continues to persist in the acute stroke population despite recent advancements in technology aimed to reduce morbidity and mortality post-stroke. Further research is required to establish protocols for management of dysphagia post-reperfusion therapies.

## Introduction

Oropharyngeal dysphagia is a well-documented consequence of acute ischaemic stroke (AIS) and is reported to affect 37–78% of patients admitted for treatment [1, 2]. Associated with aspiration pneumonia, malnutrition, dehydration, and death, dysphagia is also known to increase hospital length of stay and overall healthcare costs [3]. Management of dysphagia is primarily conducted by speech-language pathologists (SLPs) in consultation with medical officers and other members of the multi-disciplinary team (MDT) including nursing staff, dietitians, and physiotherapists [4].

Advancements in a variety of treatment options for AIS, collectively known as reperfusion therapies, have resulted in improved functional outcomes for patients post-stroke when compared to traditional treatment [5]. Reperfusion therapies include thrombolysis and endovascular thrombectomy (EVT) and aim to restore brain perfusion/recanalize brain tissue [5]. Evidence suggests that administration of thrombolysis (i.e. a pharmacological agent used to dissolve the blockage) shortly after stroke may reduce neurological damage and significantly improve clinical outcomes [6]. Like thrombolysis, EVT (i.e. removal of the clot via suctioning and/or a stent) has shown improved neurological outcomes and may be used in isolation, or in combination with thrombolysis [5]. General outcomes following reperfusion therapies can be described using a neuro-imaging scale known as “modified Thrombolysis in Cerebral Infarction” (mTICI) score, with mTICI 3 = total reperfusion, 2c = near complete reperfusion with some reduction in flow or remaining distal emboli in the cortex, 2b = partial reperfusion of equal to or greater than 50%, 2a = from partial reperfusion of less than 50%, and 1 = nil restoration of blood flow [7]. A systematic review of 10 randomised controlled trials examining the efficacy of EVT compared to usual care found moderate to high quality evidence suggesting that EVT improves functional outcomes at 90 days post-stroke [8].

Whilst research has typically focussed on general/functional outcome measures following reperfusion therapies including modified Rankin Scale (mRS), National Institutes of Health Stroke Scale (NIHSS), and mortality rates, there is limited research describing the effects of reperfusion therapies on dysphagia [9]. A 2021 retrospective chart review in Australia found 50.26% of patients (*n* = 97/193) presented with dysphagia on initial clinical swallowing examination by SLPs [10]. The study was based on clinical diagnoses and recommendations for modified diet and fluids as per SLP findings at the bedside. Following this, a 2022 single-site German study investigated dysphagia post-EVT via Fiberoptic Endoscopic Evaluation of Swallowing (FEES). The study included patients who were identified as at risk of dysphagia (failure of a water swallowing test, moderate dysarthria/aphasia, facial palsy, and NIHSS score of five or greater) [11]. Of the patients who were identified as at risk, n = 54/89 underwent FEES within 24 h, with a second FEES conducted following 72 h for patients who presented with dysphagia in the initial FEES only (*n* = 41) [11]. For patients in the initial FEES group, 90.7% (*n* = 49/52) presented with dysphagia, and laryngeal injury was detected in all patients. In the follow up (at least 72 h post) FEES group (*n* = 41), 34 patients presented with ongoing dysphagia (82.93%) [11]. As this study explored dysphagia in the at-risk group only, further multi-site studies are required to determine overall incidence rates amongst patients across multiple timepoints following reperfusion therapies, including bridging the gap between dysphagia identified within the first 24 h and its progress to after 72 h following reperfusion therapies.

Due to the limited understanding of the continuum of dysphagia recovery following EVT and thrombolysis, management pathways for dysphagia are unclear for SLPs and the wider medical team. A 2022 mixed-methods survey of 62 Australian SLPs found inconsistencies in the management of dysphagia following reperfusion therapies across Australia, with SLPs also reporting various changes in dysphagia presentation in the acute stage post-stroke [12]. Dysphagia was reported to be more fluctuant following EVT and/or thrombolysis, resulting in increased need for repeat clinical swallowing assessment, as well as a reduced incidence rates of dysphagia of a moderate severity [12]. Dysphagia was reported to be severe in cases of unsuccessful reperfusion therapies. No clear guidelines supporting the timing of dysphagia screening and assessment in patients receiving reperfusion therapies for AIS were identified and SLPs completed dysphagia assessment as early as during administration of IV thrombolysis to anywhere up to 24 h post-reperfusion therapies [12]. Other issues raised included changes in workload demands with fluctuant patient numbers and acuity due to high rates of interhospital transfers to and from EVT centres. SLPs also reported an increased need for repetition of clinical swallowing examinations and completion of thorough handovers to transfer sites [12].

As a result of limited research in the area, it is difficult to hypothesise factors that may increase dysphagia risk for this specific patient population. A 2023 systematic review found that whilst the use of reperfusion therapies is increasing, EVT and/or thrombolysis was not identified as a significant predictor of dysphagia recovery and further research in this area was recommended [13]. Additionally, a 2022 meta-analysis also found no single risk factor was independently associated with the presence of dysphagia following EVT on initial FEES (including age, sex, lesion hemisphere, NIHSS score) [11].

Without an evidence base on which to guide dysphagia management and rehabilitation post-stroke, SLPs and the wider medical team are hindered in patient prioritisation and planning. The aims of the current research were therefore to (a) explore dysphagia presentation in the acute phase (0–72 h) following reperfusion therapies, and (b) suggest comparisons of dysphagia presentation between types of reperfusion therapies (thrombolysis and/or EVT) in the acute phase of stroke recovery. Based on studies identifying general outcomes post-EVT and thrombolysis, it was hypothesised that participants with successful reperfusion therapies could present with low rates of impaired swallow function, whilst patients with unsuccessful reperfusion therapies could demonstrate increased rates of impaired swallow function.

## Method

### Participants and Study Design

Participants were prospectively recruited from two quaternary hospitals offering both EVT and thrombolysis in Queensland, Australia. Patients underwent EVT and thrombolysis at each facility according to guidelines and stroke neurologist/interventional radiologist decision making. Eligibility for reperfusion therapies in Queensland, Australia, is generally considered as: (a) age over 18 years (assessed on a case-by-case basis for those below 18) and (b) previously independent (those somewhat dependent considered on a case-by-case basis). Similarly, patients are generally considered ineligible if they present with poor prognosis as a result of premorbid conditions (i.e. metastatic cancer), minor stroke with NIHSS below four, or severe stroke with NIHSS over 25 [14]. Potential participants were identified by the researcher, SLPs or stroke nurses by regular screening of electronic patient management systems from April 2022 to December 2022. Participants were eligible to participate in the study if they presented with (a) AIS (as diagnosed by neurologist or physician and confirmed by MRI or computed tomography (CT) scan); (b) were treated with EVT, thrombolysis, or both; and (c) were aged 18 years or above. Exclusion criteria included the presence of (a) any other neurological disease or disorder, (b) severe mental illness, (c) dementia, (d) head trauma, (e) cerebral tumour or abscesses, and (f) pre-existing dysphagia. A control group was not recruited due to risk of bias associated with ineligibility for reperfusion therapies including longer times to hospital presentation, poorer functional baseline and more severe stroke.

Potential participants were screened for eligibility criteria, and if suitable, consent to contact was obtained by SLPs or nursing staff prior to initial contact by the researcher (a qualified SLP with experience in stroke). Informed consent (verbal and written) was sought from either the patient or substitute decision maker on a case-by-case basis as per medical team and SLP assessment regarding communication function, cognition, levels of alertness, and appropriateness to participate. Study information was provided in an aphasia friendly format.

The recruitment process was generally completed within the first 24 h following reperfusion therapies. However, for patients with poor levels of alertness or those deemed too medically unwell according to the medical team, recruitment was able to be completed within 48 h and data was retrospectively collected for the initial 24-h timepoint. This involved completion of the first element of the Gugging Swallowing Screen (GUSS) which assessed basic levels of alertness, saliva swallows, and volitional cough/throat clear as per documentation within the medical chart, according to screener instructions [15]. If patients met the above criteria, they subsequently failed the first part of the screener for the first timepoint (alertness, presence of voluntary cough and throat clear).

### Dysphagia Screening and Assessment

All screening and assessments were conducted by a qualified SLP with experience in post-stroke dysphagia management. The GUSS is a reliable screening test designed to identify dysphagia and risk of aspiration in the post-stroke population [15]. The GUSS was administered at three timepoints: day one (D1; 0–24 h post-reperfusion therapies), day two (D2; 24–48 h post-reperfusion therapies), and day three (D3; 48–72 h post-reperfusion therapies. The GUSS consists of two parts: (1) preliminary assessment (observations of level of alertness, presence of a voluntary cough/throat clear, saliva swallow, anterior spill of saliva, and quality of voice, i.e. “hoarse, gurgly, coated, weak”) and (2) direct swallowing tests. Patients must score 100% in the preliminary assessment (Part 1) to progress to the direct swallowing test (Part 2). The direct swallowing test involved a trial of International Dysphagia Diet Standardisation Initiative (IDDSI; 14) extremely thick fluids (“pudding consistency”) and progressed to trials of thin (regular) fluids and plain, white bread if scores of 100% were obtained (swallow present, nil cough, nil drooling, nil voice change). The final score is a sum of Parts 1 and 2 out of 20, resulting in a severity rating, with 0–9 = severe dysphagia with high risk of aspiration, 10–14 = moderate dysphagia with risk of aspiration, 15–19 = slight dysphagia with low risk of aspiration, and 20/20 = slight/no dysphagia with minimal risk of aspiration.

For quality assurance purposes and assessment of fidelity, video recordings were completed for 25% patients who progressed beyond Phase 1 of the GUSS (*n* = 3/12) and scored by a second member of the research team (EF). There was 100% agreement between the researchers, with nil revisions to scoring. Patients who were medically unwell or not alert enough to complete the assessment tasks were not video recorded.

### Stroke Variables

Information was extracted from the medical chart at each timepoint, including neuro-imaging results, EVT procedural information, functional outcomes (including NIHSS where possible), enteral feeding status, and clinical swallowing examination data including diet/fluid recommendations according to IDDSI [16] and Functional Oral Intake Scale (FOIS) [17]. Information pertaining to success of reperfusion therapy via the mTICI score was collected as per Interventional Radiology (IR) and stroke physician notes in the medical chart for patients following EVT. NIHSS score was collected from medical charts for patients prior to administration of reperfusion therapies and was reported by emergency department medical or stroke staff. NIHSS score post-treatment was not routinely reported and is therefore not present in data analysis. For analysis, NIHSS score of 0 was considered no stroke, 1–4 was considered a minor stroke, 5–15 was considered moderate stroke, 16–20 was considered a moderate/severe stroke and 21–42 was considered a severe stroke [18].

### Data Analysis

Extracted data were stored using Microsoft Excel. Median and interquartile ranges for demographic and continuous data were calculated via descriptive statistics. Data were reviewed for skewedness and kurtosis and non-parametric tests were subsequently completed. Friedman’s test was conducted to determine differences between groups over time and Spearman’s correlation was used to determine relationships between dysphagia presentation and stroke features such as NIHSS on arrival and core infarct and penumbra size. Wilcoxon Signed Ranks Test was also used to compare the relationship between NIHSS on arrival and NGT use, as well as mTICI score and dysphagia severity and Mann–Whitney Testing was used to compare treatment groups. Analyses were performed using SPSS software for Windows version 28.0 (IBM Co., Armonk, NY, USA).

## Results

### Study Population

**Fig. 1:**
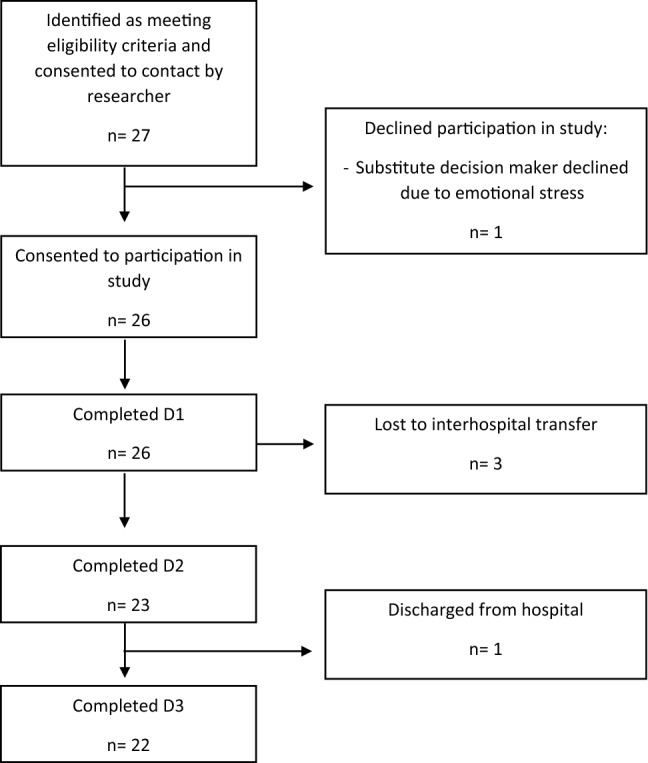
Recruitment flow chart from consent to contact to completion of the study

Twenty-seven patients were approached following consent to contact and a total of 26 participants were successfully recruited to the study. Three participants were lost to interhospital transfer and one participant was discharged from hospital prior to D3. See Fig. 1 for recruitment flow chart.

Nine females and 17 males were recruited to the study with a median age of 72 years (range = 66.75–79.50). All participants were reported as functionally independent with nil previous dysphagia prior to stroke as per eligibility criteria. Right sided infarcts were most common (*n* = 16) followed by left sided (*n* = 10) and cerebellar infarcts (*n* = 1). Nil participants required a tracheostomy. See Table 1 for further details and an overview of patient demographics and stroke characteristics.

**Table 1:** Participant demographics and stroke outcomes

Demographics	EVT	Thrombolysis	EVT & Thrombolysis	All groups
No. of participants (%total study)	11 (42.31%)	8 (30.77%)	7 (26.92%)	26 (100%)
Age, y (median, IQR)	74.73 (72, 19)	71.63 (72.5, 7.5)	73.14 (68, 17)	73.35 (72, 12)
Sex (% female)	5 (45.45%)	2 (25%)	2 (28.57%)	9 (35.61)
Left sided infarct	5 (45.45%)	3 (37.5%)	2 (28.57%)	10 (38.46%)
Right sided infarct	6 (54.55%)	5 (62.5%)	5 (71.43)	16 (61.54%)
Cerebellar involvement	0	1 (12.5%)	0	0
Core size (median, IQR)*	–	–	–	19.00 (8.25–32.75)
Penumbra size (median, IQR)*	–	–	–	17.00 (18.72–111.00)
NIHSS on arrival (mean ± SD)	12.18 (± 5.27)	8.5 (± 6.20)	16.14 (± 5.33)	12.12 (± 6.30)
Intracranial haemorrhage post-therapy (%)	0	0	2 (28.57%)	2 (7.79%)
Nasogastric tube (%)	6 (54.55%)	1 (12.5%)	3 (42.86%)	10 (38.46%)
ICU admission (%)	1	0	0	1 (3.85%)
Tracheostomy (%)	0	0	0	0

ICU admission refers to admission to ICU because of complications post-stroke and excludes routine admission to high dependency unit following EVT for observation

*Core size and penumbra size are reported for 22 patients as data were missing for four participants

*EVT* Endovascular thrombectomy, *IQR* interquartile range, *NIHSS* National Institutes of Health Stroke Scale, *ICU* Intensive care unit

### The Progression of Dysphagia According to GUSS Score (EVT vs Thrombolysis vs Both)

**Fig. 2:**
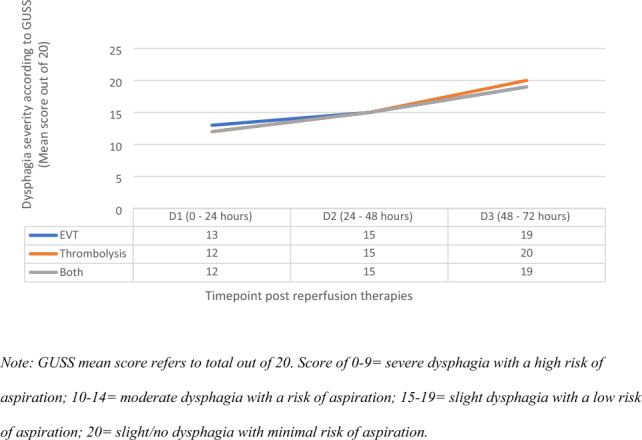
GUSS results stratified by reperfusion therapy type across three timepoints. GUSS mean score refers to total out of 20. Score of 0–9 = severe dysphagia with a high risk of aspiration; 10–14 = moderate dysphagia with a risk of aspiration; 15–19 = slight dysphagia with a low risk of aspiration; 20 = slight/no dysphagia with minimal risk of aspiration

Dysphagia severity in data analysis refers to groupings of GUSS scores between 0 and 9 for severe dysphagia, 10–14 for moderate dysphagia, 15–19 for slight dysphagia, and 20/20 for slight/no dysphagia according to GUSS protocol [15]. See Fig. 2 for the progression of dysphagia from severe to nil according to GUSS score at each timepoint.

For patients who received EVT only (*n* = 11), eight presented with severe dysphagia in D1. By D2, *n* = 4/10 of participants remaining in hospital had demonstrated improved severity category on the GUSS, and one patient had further improved by D3 (in addition to a further loss to follow up). No participants in the EVT group demonstrated a deterioration in swallow function across the timepoints.

For patients who received thrombolysis only (*n* = 8), two presented with severe dysphagia on D1. One patient improved in severity category by D2. Two patients demonstrated a deterioration in swallow function across the timepoints. One did not improve in GUSS score across the three timepoints.

Of those who received both EVT and thrombolysis (*n* = 7), five presented with severe dysphagia on D1. One patient improved in severity category by D2, with nil further changes by D3. No patients demonstrated a deterioration in swallow function across the timepoints.

A Friedman Test was conducted to examine whether there was a change in dysphagia severity at each milestone (D1, D2, D3) according to total GUSS score. There was a statistically significant difference in dysphagia severity at each timepoint (*X*^2^ = 17.22, *p* =  < 0.001), with reduction in median severity from severe to moderate between D1 and D2, and a reduction within the moderate category from D2 to D3.

Mann–Whitney Tests were conducted to determine differences in dysphagia outcomes across treatment types (EVT only, thrombolysis only, and those who received both) according to GUSS score. Dysphagia severity in the first 24 h was significantly higher for patients who received thrombolysis only compared to patients who received both therapies (*U* = 9.00,* p* = 0.024). Additionally, dysphagia severity in the first 24 h was significantly higher for patients who received thrombolysis only compared to patients who received EVT only (*U* = 15.50, *p* = 0.016). There was no significant difference in dysphagia presentation between patients post-EVT and patients who received both therapies (*p* = 0.70). See Fig. 2 for a visual representation of the data.

### Penumbra and Core Size and Dysphagia

A Spearman’s rank-order correlation was completed to determine the relationship between core infarct size and penumbra (in millilitres) and dysphagia severity. No statistically significant correlation was found between core size and dysphagia severity on D1 (*p* = 0.948), D2 (*p* = 0.526), or D3 (*p* = 0.805). Penumbra size was also not significantly correlated with dysphagia severity on D1 (*p* = 0.176), D2 (*p* = 0.384), or D3 (*p* = 0.178).

### The Relationship Between mTICI Score and Dysphagia

For patients following EVT, mTICI score was significantly negatively correlated with dysphagia at D1 (*Z* = − 3.325, *p* =  < 0.001), meaning patients with a higher mTICI score (better reperfusion outcomes) presented with lower degrees of dysphagia severity according to GUSS score. mTICI score was not significantly correlated with outcomes at D2 or D3.

### Number of Passes During EVT and Dysphagia

One pass was required for seven out of the 11 EVT patients. Of those seven, three presented with slight dysphagia and four presented with severe dysphagia according to D1 GUSS (within 24 h). By D3 (48–72 h), 2 patients were lost to interhospital transfer. The remaining five out of the seven patients improved their scores, but dysphagia did not completely resolve. Two passes were required for 2 patients out of 11 patients who received EVT. Both presented with severe dysphagia at D1 according to GUSS (100%). One patient improved to moderate dysphagia by D3, and the other remained severe. Two patients required four and six passes respectively, and both presented with severe dysphagia according to GUSS at each timepoint. The patient who required four passes died in hospital following completion of the study. The correlation between the number of passes during EVT and the severity of dysphagia was statistically significant at D1 (*p* = 0.009) and D2 (p = 0.026) and approached significance at D3 (*p* = 0.059).

### NIHSS Score and Dysphagia

**Fig. 3:**
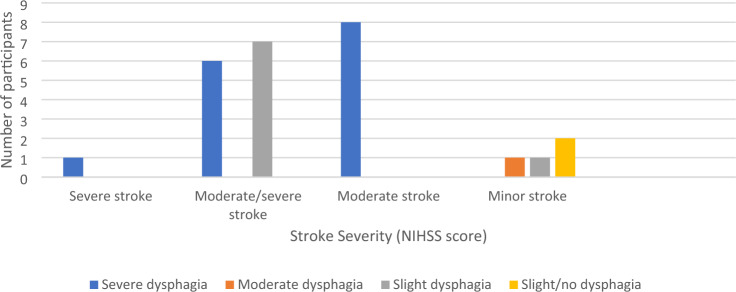
Dysphagia severity (GUSS score) 0–24 h post-reperfusion therapies stratified by stroke severity on arrival (NIHSS score)

See Fig. 3 for dysphagia severity stratified by NIHSS stroke severity. A Spearman's rank-order correlation was completed to determine the relationship between stroke severity according to NIHSS score. There was a statistically significant positive correlation between NIHSS score on arrival to hospital and severity of dysphagia according to GUSS at D1 (0–24 h post-reperfusion therapies) (*r*_*s*_ = 0.818, *p* =  < 0.001), D2 (*r*_*s*_ = 0.766, *p* =  < 0.001), and D3 (*r*_*s*_ = 0.595, *p* = 0.004).

Regarding dysphagia recovery, the patient with severe stroke presented with severe dysphagia across each timepoint. For patients with moderate/severe stroke (*n* = 8), one patient was lost to interhospital transfer following D1, one (out of the seven remaining) improved by D2, and a further three had improved by D3. For patients with moderate stroke (*n* = 13), one patient deteriorated in swallow function. For patients with minor stroke (*n* = 4), two were discharged by D2 and the remaining two patients maintained slight to no dysphagia respectively.

### Oral Feeding and Dysphagia

In the EVT only group, *n* = 6/11 were recommended to continue nil by mouth (NBM) by the treating SLP following clinical swallowing examination on D1. By D3, four of the ten remaining patients (40%) continued NBM status and the remaining three were recommended oral diets including puree, minced and moist, and soft and bite sized. Conversely, *n* = 3/11 commenced thin fluids and a regular diet on D1 with nil increase by D3.

For the thrombolysis group, *n* = 2/8 were recommended NBM by the treating SLP on D1, whilst *n* = 6/8 commenced D1 on a regular diet and thin fluids. One patient had deteriorated by D3 and resumed NBM status.

For patients who received both EVT and thrombolysis, *n* = 4/7 were recommended NBM by the treating SLP, two patients commenced a regular diet and thin fluids and the remaining patient commenced a modified diet and thin fluids on D1. The patient recommended a modified diet had been upgraded to a regular diet by D2, and one patient (originally recommended a full diet and thin fluids) regressed to a puree diet by D3.

### Non-oral (enteral) Feeding and Dysphagia

Six out of 11 patients who received EVT required enteral feeding during the first three days of their admission, compared with one out of eight patients who received thrombolysis and three out of seven who received both therapies. A Wilcoxon signed-rank test showed that NIHSS on arrival elicited a statistically significant requirement in NGT use within the first three days following reperfusion therapies across all groups (*Z* = − 4.46, *p* =  < 0.001). Subgroup analyses were not conducted due to the small sample size in each group.

## Discussion

This study is, to the authors’ knowledge, the first of its kind to track dysphagia outcomes for reperfusion therapy subtypes in the hyperacute phase across multiple timepoints using a validated and reliable clinical swallowing screening tool. The overall incidence of dysphagia in the acute stage of stroke varies between 40 and 80% in the literature [19]. In the present study, across three groups (EVT only, thrombolysis, and both) the incidence of any severity dysphagia (including slight dysphagia) within the first 24 h of reperfusion therapy was 92.31% (*n* = 24/26), 91.30% (*n* = 21/23) by 48 h, and 90.91% (*n* = 20/22) by 72 h, indicating ongoing high rates of dysphagia. Testing also found significantly higher rates of dysphagia in the thrombolysis only group compared to patients who received both therapies (*p* = 0.024) and EVT only (*p* = 0.016). The cause for high rates of dysphagia is unclear, but may be attributable to a latent effect of general anaesthetic on the swallow function (as part of the EVT procedure), the nature of referral criteria for reperfusion therapies (i.e. stroke severity), and/or the hyperacute screening period of the study (within 72 h), and should be considered with caution given the relatively small sample size.

Research describing the impact of reperfusion therapies on dysphagia outcomes in the early stages of stroke is limited. A 2022 single-site study by Lapa et al. examined 54 patients following EVT (± thrombolysis) who failed dysphagia screening via FEES within 24 h post-extubation and re-examined those with significant dysphagia via FEES within a 72-h timeframe (10). The current study included all patients following EVT and/or thrombolysis to identify potential neurological deteriorations or changes across the acute trajectory, whereas Lapa et al. included patients with significant dysphagia only in their follow up group. Additionally, the current study completed screening at three timepoints (0–24 h, 24–48 h, and 48–72 h post) to provide a detailed exploration of the change in swallow function, whereas the Lapa et al. study focussed on two timepoints (the first 24 h and following 72 h). The current study supports Lapa et al. (2022) which found 90.7% (*n* = 49/54) incidence rate of dysphagia 2–24 h following extubation post-EVT. Of those patients, 69.4% demonstrated ongoing dysphagia at the second assessment (72 h following the initial). The authors proposed that dysphagia following EVT is therefore unlikely to transient in nature or primarily related to the impact of the EVT procedure itself (including sedative effects) [9].

Differing from the Lapa et al. (2022) study, several metrics related to EVT procedure and outcomes were analysed in relation to dysphagia including number of passes (attempts at retrieval of the clot), penumbra and core size in the present study. Research has previously demonstrated worsened functional outcomes with increased number of passes during the retrieval procedure [20]. This may be related to increased risk of symptomatic intracranial haemorrhage or weakening of the vessel involved. Also known as the first pass effect, reperfusion following one attempt at clearance via EVT is associated with improved clinical outcomes [21]. This current study supported these findings, with the largest improvement in dysphagia across each timepoint observed in patients who received one pass, whilst severe dysphagia at each timepoint and death occurred for the two patients who received four to six passes respectively. Due to the small sample size, further research is required to explore the relationship between attempts at retrieval during EVT and dysphagia outcomes which may act as a predictive factor to guide swallowing screening and dysphagia management.

Previous research has also investigated the impacts of penumbra size on functional recovery. Chen et al. (2017) found the odds of good clinical outcome at 3 months post-stroke increased by 7.4% for every 1% of penumbra salvaged [22]. However, nil research has been identified exploring the relationship between core and penumbra size on dysphagia. The current study did not identify a significant correlation between penumbra or core size in millilitres and dysphagia severity. However, authors suggest that this may be due to a small sample size, or the importance of lesion location in addition to amount neurological cell death as indicator of dysphagia. This may suggest that patients who appear to represent a clinically small core infarct size should still be considered at risk of dysphagia.

Whilst core and penumbra size were not significantly correlated with severity of dysphagia in the current study, the severity of stroke according to NIHSS score is a known predictor of functional outcomes and dysphagia. NIHSS scores pre-intervention (i.e. on arrival to hospital) were significantly related to dysphagia outcomes for both ordinal values and when collated into severity categories in the current study. NIHSS scores post-treatment were not routinely reported and therefore not part of analysis. This study found that as NIHSS score increased (i.e. as severity of stroke increased), so too did the severity of dysphagia. This is information may be useful to SLPs, nursing staff and medical teams in triaging and planning for dysphagia management as relationships between NIHSS score and subsequent deficits can persist beyond administration of reperfusion therapies. Previous research has supported the use of NIHSS on arrival as a predictive factor for dysphagia, with the correlation between NIHSS scores above 5 and the presence of dysphagia reported in 2017 [23]. Whilst representing a small sample size, this is supported within the current study, as 100% of patients with NIHSS scores above 5 (*n* = 22) presented with dysphagia.

Another measure of success for functional outcomes following reperfusion therapies well documented within the literature is the use of mTICI score. The present study found a statistically significant correlation between mTICI score following EVT and dysphagia severity according to GUSS score for the first timepoint only. mTICI score was not routinely reported following thrombolysis at the sites included in the study. This result is supported widely by previous research focussing on the relationships between NIHSS score, mRS and mTICI scores post-intervention [24]. Whilst dysphagia is not distinctly part of NIHSS or mRS scoring, the NIHSS includes facial palsy and dysarthria as key parameters, which are known predictors of dysphagia [25]. It is therefore reasonable to assume that improved outcomes on NIHSS and mRS scales with a known correlation to mTICI scores could also be reflective of dysphagia outcomes. SLPs and medical teams may therefore consider mTICI scores in triaging and managing patients with suspected dysphagia, as the present study found that patients with higher mTICI scores were less likely to present with swallowing difficulties with 0–24 h post-EVT.

In addition, the use of enteral feeding related to dysphagia post-stroke has been widely described in the literature. In the acute stage, approximately 20% of patients post-stroke are reported to require enteral feeding via NGT [26]. The current study found a slightly higher incidence rate, with over one third of patients receiving nutrition via NGT within the first 72 h of admission. These results are similar to those identified in a retrospective chart review by Minchell et al. (2022), which found over a quarter of patients (25.4%) required enteral feeding following reperfusion therapies. Previous research also by Minchell et al. (2022) found a lack of consistency in the use of dysphagia related NGT insertion following reperfusion therapies [12]. Some sites were reported to insert the NGT during the administration of reperfusion therapy, following failure of a nursing led screening tool, or following monitoring of dysphagia and oral intake. NGT insertion has previously been reported to increase bleeding risk due to the physiological nature of thrombolysis, with several guidelines recommending withholding insertion until over 24 h post-therapy [27, 28]. With ongoing high NGT use post-reperfusion therapies demonstrated during this study and the Minchell et al. (2022) review, SLPs, dietitians, and the wider MDT may benefit from an evidence base to assist with enteral feeding protocols for patients post-reperfusion therapies.

## Clinical Implications

Whilst presenting a relatively small sample size, results of this study may hold implications for clinical practice if confirmed in larger datasets. Clinicians could (a) consider stroke location, rather than penumbra or core size, when triaging dysphagia post-reperfusion therapies; (b) consider increased risk of severe dysphagia for patients who receive thrombolysis only; (c) consider successful mTICI scores (mTICI 3) and fewer passes during EVT to be indicative of a lower likelihood of dysphagia; (d) continue to consider higher NIHSS scores as an indicator of potential dysphagia, and (e) continue to advocate for the importance of ongoing SLP input in post-stroke care with persistent high incidence rates of dysphagia (> 90%) across all groups.

## Limitations

Whilst this study was conducted at two quaternary sites offering EVT in Queensland, Australia, the authors acknowledge that reperfusion therapies are used internationally with varying protocols, techniques and retrieval devices, and results may therefore not be reflective of all practice. The authors also acknowledge limitations associated with clinical dysphagia screening and assessment without the use of instrumental swallowing studies. However, dysphagia screening and clinical swallowing examinations are standard practice in Australia where patients do not typically undergo instrumental assessments in the hyperacute stage of stroke (i.e. within the timeframe of the current study) [29]. In the early stages post-stroke, diet and fluid recommendations are usually determined as per least risk identified during clinical swallowing examination by a SLP, with instrumental assessments conducted in cases of persisting dysphagia or on a case-by-case basis [29]. This study utilised validated and widely used screening tools and assessments within the acute stroke population.

A further limitation of this study is the lack of power due to small sample size. Statistical analyses should therefore be interpreted with caution. Due to the hyperacute and multi-site nature of the study, it was not possible to recruit all patients who met eligibility criteria or to monitor the details of ineligible participants. Patients frequently transfer to EVT centres hospital sites for treatment (and sometimes may be deemed ineligible following transfer) and are transferred to their referring hospital as soon as feasible and safe. Finally, this study was planned and conducted at designated COVID-19 hospitals which underwent research closures in 2020 and 2021, resulting in delays to study commencement and therefore reduced participant numbers.

## Conclusion

This multi-site, prospective pilot study aimed to explore the incidence and trajectory of dysphagia in the acute stage post-reperfusion therapies (EVT ± thrombolysis) with use of a reliable post-stroke screening tool to provide patients, SLPs, and medical teams with an evidence base to guide post-stroke care. Dysphagia screening across three timepoints (0–24, 24–48, and 48–72 h) identified ongoing high levels of dysphagia, with some improvement in screening scores for all groups (EVT, thrombolysis, and patients who received both). The number of passes during EVT, NIHSS on arrival, and mTICI score were significantly correlated with dysphagia outcomes, whereas penumbra and core size were not. Severity of dysphagia was higher in the thrombolysis only group compared to patients who received EVT or both therapies. Due to the small sample size, additional large-scale studies are required to develop evidence-based dysphagia management protocols (including updated guidelines for enteral feeding).

## Data Availability

The data are not publicly available due to data containing information that could compromise research participant privacy/consent.
